# Supplementary material for: Sustained-input switches for transcription factors and microRNAs are central building blocks of eukaryotic gene circuits
Source: Genome Biol. 2013 Aug 23;14(8):R85. doi: 10.1186/gb-2013-14-8-r85 (PMC4054853; doi:10.1186/gb-2013-14-8-r85)
Supplement: Additional file 5 — HTML Browsable Motif Output. Zipped folder containing all WaRSwap and FANMOD motif output, viewable in a web browser. [file gb-2013-14-8-r85-S5.ZIP › HTML_browsable_motif_output/FANMOD_ath_tair9/sigs_fanmodm-2000.pvals.heatmaps.html/motif_id_46_011101001_tftype_ath_upstream_-1000_0.html]

```
BG_MODEL = FANMOD
MOTIF_ID = 46_011101001
TF_TYPE = ath
UPSTREAM = -1000_0


PVals
FN_0.2	FN_0.4	FN_0.6	FN_0.8
dg_60.genes	0.015	0.001	0.809	0.491
dg_70.genes	0.012	0.003	0.813	0.48
dg_80.genes	0.013	0.002	0.804	0.516

ZScores
FN_0.2	FN_0.4	FN_0.6	FN_0.8
dg_60.genes	2.184	2.865	-0.924	-0.804
dg_70.genes	2.216	2.938	-0.918	-0.786
dg_80.genes	2.211	2.98	-0.89	-0.825

StDevs
FN_0.2	FN_0.4	FN_0.6	FN_0.8
dg_60.genes	218.837	129.971	23.265	4.698
dg_70.genes	216.488	126.495	23.699	4.664
dg_80.genes	213.366	127.617	24.101	4.952
```
